# On the Diffraction Limit for Lensless Imaging

**DOI:** 10.6028/jres.104.029

**Published:** 1999-10-01

**Authors:** Klaus D. Mielenz

**Affiliations:** Oakland, MD 21550

**Keywords:** aperture diameter, diffraction, Fraunhofer-Airy profile, Fresnel-Lommel theory, image size, irradiance, lensless imaging, Petzval, pinhole photography, radiant flux, Rayleigh, resolution, Sparrow criterion

## Abstract

The diffraction limit for lensless imaging, defined as the sharpest possible point image obtainable with a pinhole aperture, is analyzed and compared to the corresponding limit for imaging with lenses by means of theoretical considerations and numerical computations using the Fresnel-Lommel diffraction theory for circular apertures. The numerical result (*u* = π) obtained for the best configuration parameter *u* which defines the optical setup is consistent with the quarter-wave criterion, and is the same as the value reported in a classical paper by Petzval but smaller than the value (*u* = 1.8π) found by Lord Rayleigh. The smallest discernible detail (pixel) in a composite image is defined by an expression found by Rayleigh on applying the half-wave criterion and is shown to be consistent with the Sparrow criterion of resolution. The numerical values of other measures of image size are reported and compared to equivalent parameters of the Fraunhofer-Airy profile that governs imaging with lenses.

## 1. Introduction

**Fig. 1:**
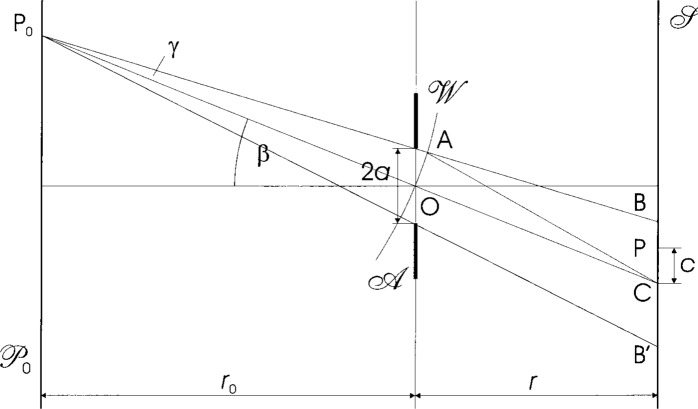
Lensless imaging by a circular aperture A of diameter 2*a*. $P_{0}=\text{object plane}$, P_0_ = object point, O = aperture center, S=screen, BPCB’ = geometrical image, *c* = *PC* = radial distance from image center.

Lensless imaging is known as the basis of the camera obscura, or pinhole imagery. It has modern technical and scientific applications where the use of lenses is not possible (e.g., x-ray optics) or must be avoided (e.g., illumination of absolute radiometers). The underlying concept is illustrated in Fig. 1: A small circular aperture A limits the pencil of rays proceeding from a luminous point P_0_ in an object plane $P_{0}$ and, thus, produces an image in the form of a small patch of light BPCB’ on a screen S. It is customary to describe this image in terms of a dimensionless image coordinate *v* and configuration parameter *u* defined as

$$v=\frac{2\pi ac}{\lambda r},\;u=\frac{2\pi a^{2}(r_{0}+r)}{\lambda r_{0}r},$$ (1)

where *λ* is the wavelength of light, *a* is the radius of the aperture, *r*_0_ and *r* are the object and image distances shown in the figure,^1^ O is the aperture center, A is a point on the aperture rim, and *c* = *CP* is the radial distance from the image center C. As it is the object of this paper to assess the actual width of the image produced by an aperture of given radius *a*, we will use $v/\sqrt{u}$ instead of *v* as the image coordinate because it is proportional to *c* but independent of *a*.

**Fig. 2:**
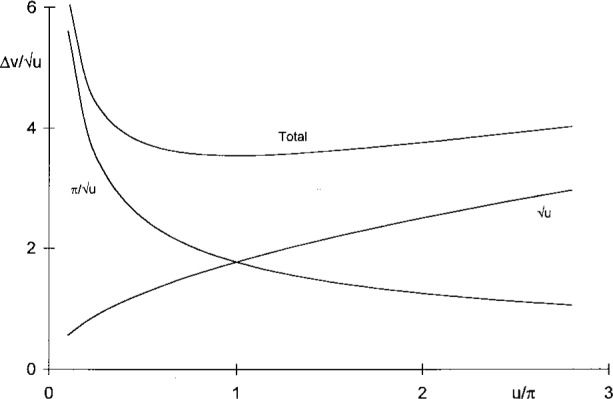
Petzval’s estimate. The diffraction limit occurs at the intersection, *u* = π, of the image widths due to diffraction (left) and geometrical optics (right).

A general idea of the nature of the diffraction limit for lensless imaging can be obtained from a classical paper by Petzval [1], who assumed that for *r*_0_ >> *r* every image point P is spread by Fraunhofer diffraction and thus has a finite width on the order of 2*λr*/*a*. The aggregate image width 2Δ*c* exceeds the geometrical width 2*a* by the same amount so that Δ*c* = *a* + *λr*/*a*, or $\Delta v/\sqrt{u}$ as shown by the upper curve in Fig. 2. This total image radius has a minimum for $\sqrt{u}=\pi /\sqrt{u}$ so that

$$\begin{array}{c} {\left(u\right)}_{\text{Petzval}}=\pi ,\;{\left(\Delta v/\sqrt{u}\right)}_{\text{Petzval}}=2\sqrt{\pi },\\ {\left(2a\right)}_{\text{Petzval}}=\sqrt{2\lambda r},\;r_{0}>>r, \end{array}$$ (2)

where (2*a*)_Petzval_ is the aperture diameter expected to give the sharpest image. Petzval’s analysis is admirable on account of its simplicity, although the Fraunhofer approximation is not applicable for lensless imaging and the incoherent superposition of elementary diffraction patterns is inadmissible.

It is easy to show that Petzval’s result for *u* is consistent with the quarter-wave criterion of resolution. Let A be a point located on the spherical wavefront W incident on the aperture and on a marginal ray passing at the aperture center, so that

$$AC^{2}=P_{0}A^{2}+P_{0}C^{2}-2P_{0}AP_{0}C\cos\gamma ,$$ (3a)

as may be seen by applying the cosine theorem to the triangle *P*_0_*AC*. In the paraxial approximation cos*β* ~ 1 one finds *P*_0_*A* = *P*_0_*O* ~ *r*_0_ and *AC* ~ *r* + Δ*r*, where Δ*r* is the small path difference *AC − OC*. Therefore,

$$\Delta r\approx \frac{r_{0}(r_{0}+r)(1-\cos\gamma )}{r}\approx \frac{a^{2}(r_{0}+r)}{2r_{0}r}=\frac{\lambda u}{4\pi },$$ (3b)

where it was assumed that Δ*r*^2^ << *r*^2^ and *γ* was evaluated as the small angle *a*/*r*_0_. This proves that the quarter-wave criterion is satisfied when *u* = π.

A further refinement of the theoretical treatment requires the Fresnel-Lommel equations [2] which govern the diffraction effects of lensless imaging,

$$E(u,v)=E_{\text{geom}}(v){\left|\alpha (u,v)\right|}^{2},\;E_{\text{geom}}(v)=\frac{\Phi_{0}{r_{0}}^{2}}{\pi a^{2}{\left(r_{0}+r\right)}^{2}},$$ (4a)

$${\left|\alpha (u,v)\right|}^{2}=\frac{u^{2}}{4}[L^{2}(u,v)+M^{2}(u,v)],$$ (4b)

where *E*(*u*,*v*) is the irradiance at the point P of Fig. 1, *E*_geom_(*v*) is the geometrical irradiance in the absence of diffraction, *Φ*_0_ is the radiant flux admitted by the aperture, |*α* (*u*,*v*)|^2^ is the modification of *E*_geom_(*v*) by diffraction, and L(*u*,*v*) and M(*u*,*v*) are functions defined by Lommel as linear combinations of infinite series of Bessel functions.

**Fig. 3:**
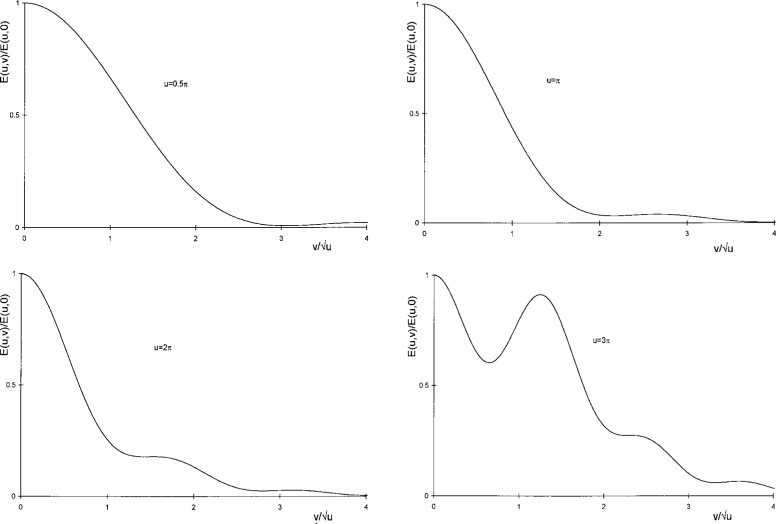
Normalized Fresnel-Lommel diffraction profiles for *u* = 0.5 π, π, 2π, and 3π.

In the past, computations based on these equations were tedious but nonetheless Lommel provided numerical tables of *E*(*u*,*v*) and Rayleigh [3] used these tables for a further analysis of pinhole imaging. Rayleigh plotted the diffraction profiles which are reproduced here as Fig. 3 and, without additional calculations, judged that “*u* = ½π *is too large and u = 3*π *is too great. The only question that can arise is between u* = π *and u = 2*π. *The latter has decidedly the higher resolving power, but the advantage is to some extent paid for in the greater diffusion of light outside the image proper.*” He conducted visual and photographic experiments to settle this question and found that the sharpest images were obtained for

$$u_{\text{Rayleigh}}=1.8\pi ,\;{\left(2a\right)}_{\text{Rayleigh}}=\sqrt{3.6\lambda r_{0}r/(r_{0}+r)}.$$ (5)

Rayleigh did not explicitly state the image size corresponding to this value of *u*. However, he showed that, for any aperture diameter 2*a* and in the limit *r*_0_ >> *r*, the greatest path difference at the point P in Fig. 1 is 2*ac*/*r*, so that “*the illumination will not be greatly reduced until the extreme discrepancy of phase reaches half a wavelength*.” This gives 2Δ*c* = *λr*/2*a* or Δ*v* = 0.5π, which turns out to be an excellent estimate of the onset of resolution in the sense of the Sparrow criterion [4] because the relative central irradiance^2^ in the composite pattern of two Lommel profiles separated by $2\Delta v/\sqrt{u}=\pi /\sqrt{u}$,

$$\frac{E_{\min}}{E_{\max}}=\frac{2E(u,\Delta v/\sqrt{u})}{1+E(u,2\Delta v/\sqrt{u})},$$ (6a)

is 0.9974 for *u* = π, 0.9967 for *u* = 1.8π, and 1.0086 for the Airy profile defined by Eq. (7a), below. Accordingly, the smallest discernible detail (pixel) in a composite image is given by

$$\Delta v_{\text{Sparrow}}=0.5\pi .$$ (6b)

## 2. Analysis

As the use of Lommel’s equations [Eqs. (4a,b)] for numerical computations is no longer a problem, it is the purpose of this paper to re-examine the question of an optimal aperture diameter for lensless imaging and to characterize the resulting image distribution of irradiance and radiant flux in quantitative terms. This work was performed using the algorithms for Fresnel diffraction published in Ref. [5], which should be consulted for mathematical and computational details. Readers wishing to perform computations may also consult a recent paper on a similar topic by Shirley [6].

When a lens is placed at the aperture A in Fig. 1 and $P_{0}$ and S are conjugate object and image planes, the irradiance distribution in the diffraction pattern will be given by the Fraunhofer-Airy formula,

$$E(v)=\frac{\Phi_{0}\pi a^{2}}{\lambda^{2}r^{2}}{\left[\frac{2J_{1}(v)}{v}\right]}^{2},$$ (7a)

where J_1_ is the first-order Bessel function of the first kind and the other quantities are the same as above. As this equation also applies to Fresnel-Lommel diffraction in the limit *u* → 0,^3^ it will be useful to analyze the profiles in Fig. 3 by criteria which are equivalent to accepted criteria for assessing lens images. The properties of the Airy function [Eq. (7a)] that will be used for this purpose are listed in Table 1, where the values in the last column represent the fractions of the total flux *Φ*_0_ contained in a circle of radius Δ*v*,

$$f(\Delta v)=\frac{1}{2}\int_{0}^{\Delta v}dvv{\left[\frac{2J_{1}(v)}{v}\right]}^{2}=1-J_{0}^{2}(\Delta v)-J_{1}^{2}(\Delta v),$$ (7b)

according to a formula derived by Rayleigh in an article on the wave theory of light [6]. The best known quantity shown in Table 1 is the radius Δ*v*_Airy_ of the central maximum (Airy disk). Because 84 % of the total flux is concentrated in the central disk, it is often assumed that the diffraction pattern consists of the Airy disk alone.

**Fig. 4:**
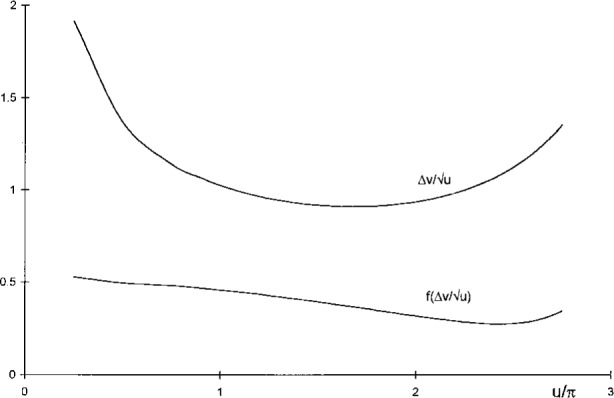
Area width $\Delta v/\sqrt{u}$ and flux fractions $f(\Delta v/\sqrt{u})$ as functions of *u*.

Figure 3 shows at a glance that width criteria based on a single irradiance value would be unreliable measures of image sharpness as they favor the central portion of the profiles and ignore the formation of a second maximum which is evident in the figures. For example, the halfwidth of the profiles decreases monotonically until, near *u* = 3π, the profile turns up before reaching the half-power point. On the other hand, the area width defined in the third row of Table 1 is quite suitable as it measures the feature which is most obvious on visual inspection of these profiles; namely, the concentration of *E*(*u*,*v*)/*E*(*u*,0) near the ordinate axis. Rayleigh may have used it intuitively when judging these profiles, and in this work it was applied mathematically by means of the quadrature formula

$$\frac{\Delta v}{\sqrt{u}}=\frac{1}{\sqrt{u}{\left|\alpha (u,0)\right|}^{2}}\int_{0}^{\infty }dv{\left|\alpha (u,v)\right|}^{2}\approx \frac{\delta v}{\sqrt{u}{\left|\alpha_{0}\right|}^{2}}\sum_{n=0}^{N}{\left|\alpha_{n}\right|}^{2},$$ (8)

which allows computations of area widths by straightforward summation of |*α_n_*|^2^ = |*α* (*u*,*v_n_*)|^2^ for equidistant arguments *v_n_* = (*n* + ½) δ*v* up to a largest value *N* for which |*α_n_*|^2^ is suitably small. The increments and upper limits used were δ*v* = 0.01 *u* and *N* = 500, resulting in numerical values accurate to six digits or better. As shown in Fig. 4, these computations revealed the existence of a shallow minimum near

$$u=1.7\pi ,\;\Delta v/\sqrt{u}=0.910\;925,$$ (9)

which differs insignificantly from the value 1.8π found by Rayleigh, and thus verifies that the latter is consistent with the area width criterion.

However, what really determines the sharpness of the image is not the concentration of irradiance as plotted in Fig. 3 but the physical concentration of radiant flux in the image plane itself. This aspect was mentioned but not explored by Rayleigh, and will be considered next. The quantity required for this purpose is the fractional flux contained in a circle of radius Δ*c* = λ*r*Δ*v*/2π*a* in the image plane, as in Eq. (6b), and thus Eqs. (4a,b) were used to derive the following summation formula for Lommel profiles,

$$\begin{array}{c} f(\Delta v/\sqrt{u})=\frac{2\pi }{\Phi_{0}}\int_{0}^{\Delta c}dc\;cE(u,v)=\frac{2}{u^{2}}\int_{0}^{\Delta v}dv\;v{\left|\alpha (u,v)\right|}^{2}\\ \approx \frac{{\left(\delta v\right)}^{2}}{2u^{2}}\sum_{n=0}^{N}\left(2n+1)\{{\left|\alpha_{n}\right|}^{2}+{\left|\alpha_{n+1}\right|}^{2}\right\}, \end{array}$$ (10)

where the notation is the same as in Eq. (8) but *v* and |*α* (*u*,*v*)|^2^ are replaced by their arithmetic means for each element of summation. Equation (10) was used to generate lists, accurate to six digits, of $f(\Delta v/\sqrt{u})$ for consecutive upper limits *N* ≤ 500 and increments δ*v* = 0.01*u*. These lists were used as lookup tables, and linear interpolation was used to obtain final results for given values of *u* and $\Delta v/\sqrt{u}$.

**Fig. 5:**
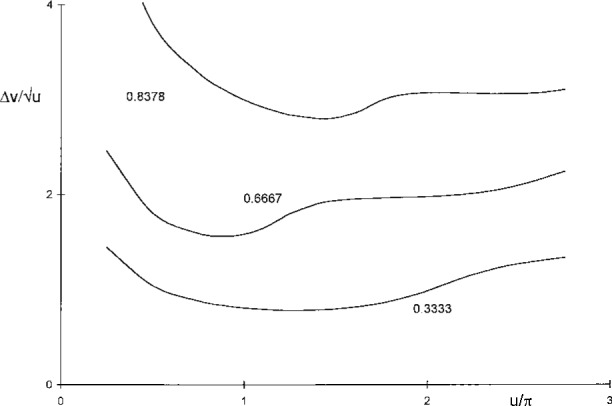
Flux widths $\Delta v/\sqrt{u}$ for $f(\Delta v/\sqrt{u}=0.3333$, 0.6667, and 0.8378.

**Fig. 6:**
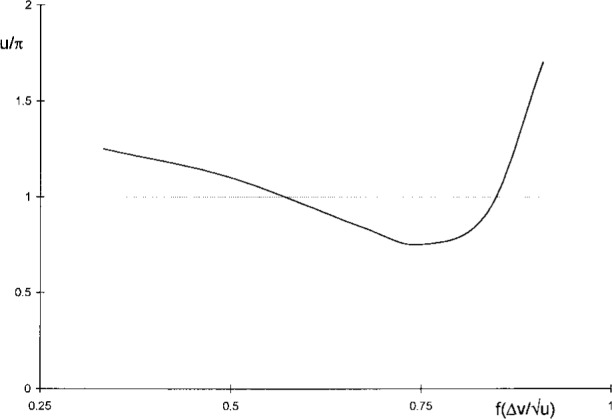
Dependence of best *u* on flux fraction $f(\Delta v/\sqrt{u})$.

The values of $f(\Delta v/\sqrt{u})$ obtained in this manner for the area widths computed earlier are plotted as the lower curve in Fig. 4. While approaching 50 % for small values of *u*, as should be expected from Table 1, these flux fractions decrease for larger values of *u* and, where $\Delta v/\sqrt{u}$ is a minimum, they are only on the order of 35 %. This was deemed insufficient for judging the profiles in their entirety. Therefore, it was decided to apply Eq. (10) in a different manner so that it would directly yield the values of *u* for which $f(\Delta v/\sqrt{u})$ is contained in the smallest possible width $\Delta v/\sqrt{u})$. The flux fractions used for these computations were 0.3333, 0.5, 0.6667, 0.75, and 0.8378, the latter being equivalent to the Airy disk in Table 1. The results obtained are illustrated in Fig. 5, showing that a shallow but discernible minimum of $\Delta v/\sqrt{u}$ exists for every $f(\Delta v/\sqrt{u})$. The values of *u* at which these minima occur are all different. However, as shown by Fig. 6, they are neatly clustered around a median value of

u=π, (11)

which was considered the best overall choice of *u* based on the concentration of radiant flux in the image.

**Fig. 7:**
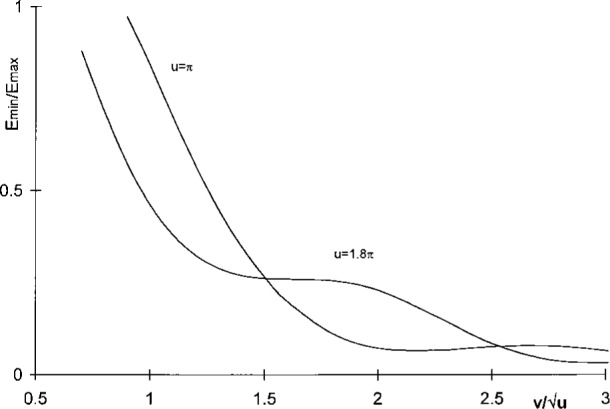
Relative central irradiance, *E*_min_/*E*_max_, in the composite pattern of two Lommel profiles separated by $2\Delta v/\sqrt{u}$ for *u* = π and 1.8π.

As a final test, we calculated the relative central irradiance using Eq. (6a) in the composite pattern of two Lommel profiles separated by given amounts $2\Delta v/\sqrt{u}$. The results obtained are plotted in Fig. 7, showing that for the usual definition of resolving power in terms of a relative central irradiance on the order of 0.7 the configuration parameter *u* = 1.8π is superior. On the other hand, the advantage lies with *u* = π when larger separations are considered, as has often been advocated.

## 3. Conclusions

On account of the complicated nature of the Lommel profiles it is no surprise that the above analysis gives no unequivocal answer regarding a best configuration parameter *u* for lensless imaging. We have verified Rayleigh’s value as a minimum of area width, confirmed Petzval’s value by computations of flux widths, and were unable to make a choice using the theory of resolution. This ambiguity can undoubtedly be attributed to the fact that the various minima of image size found in this analysis are all shallow so that, on the whole, the difference between *u* = π and 1.8π is insignificant for practical purposes. The same can be inferred from the observation made by writers on pinhole photography that, although the best aperture diameters are usually stated within 0.01 mm, deviations on the order of 0.1 mm have little effect on image quality. It appears that Rayleigh reached a similar conclusion. He mentioned that Petzval’s value, *u* = π, was quoted in a pamphlet on pinhole photography and remarked that the corresponding “*detail in a photograph … was not markedly short of that observable by direct vision.*” At the same time, he stated that images obtained for *u* = 1.8π “*fully bore out expectations.*”

It would of course be possible to compromise and adopt an intermediate best value of *u*. This seemed inadvisable as it might be misinterpreted as an improvement or refinement of the results obtained by Petzval and Rayleigh. The above analysis has shown that both are valid and that there is a fairly wide range of *u*’s which give acceptably sharp images. This is in fact a practical advantage, as it permits the use of one and the same aperture for a variety of image distances and/or wavelengths.

However, from a theoretical point of view it is desirable to have a value which is, not only consistent with experience, but also conforms to accepted criteria used elsewhere for assessing image quality. On this basis, *u* = π is preferable as it is consistent with the quarter-wave criterion whereas *u* = 1.8π is not. By the same token, the Sparrow width in Eq. (6b) is theoretically superior to the value in Eq. (2) because it conforms to the half-width criterion. Accordingly, the final result adopted in this paper for the diffraction limit of lensless imaging is

$$u=\pi ,\;\Delta v/\sqrt{u}\geq 0.5\sqrt{\pi },\;2a=\sqrt{2\lambda r_{0}r/(r_{0}+r)},$$ (12)

where $0.5\sqrt{\pi }$ is the pixel size, defined as the smallest discernible detail in accordance with the Sparrow criterion. Equation (12) should not be construed as a criticism of Rayleigh’s astute assessment of the Lommel profiles in Fig. 3. It merely ensures that the diffraction limits for imaging with and without lenses are defined consistently, but does not affect the practical aspects of Rayleigh’s work. Yet, from a historical perspective it is interesting that Rayleigh did not apply his own quarter-wave criterion to define *u* while, at the same time, he used a half-wave criterion to define the image size.

The optical and radiometric properties of the Lommel profile for *u* = π are summarized in Table 2. Four of these parameters were defined to be equivalent to the values given in Table 1 for Airy profiles. The fifth is the geometrical width $\Delta v/\sqrt{u}=\sqrt{u}$ that would be obtained in the absence of diffraction.^4^ The remaining columns of Table 2 show the numerical values of $\Delta v/\sqrt{u}$ and the corresponding relative irradiances $E_{\text{rel}}=E(u,\Delta v/\sqrt{u})/E(u,0)$, flux fractions $f(\Delta v/\sqrt{u})$, and relative central irradiance *E*_min_/*E*_max_ in a double image. The equivalent Airy disk in Table 2 is defined so that the corresponding flux fraction is the same as for the Airy profile, and the Rayleigh width is half as large;^5^ their numerical values can be closely approximated by 3 and 1.5, respectively. It should be noted that the geometrical width is twice as large as the Sparrow width and appears near the bottom of the table. This shows that, on the whole, the diffraction-limited image is sharper than the geometrical image.

## Figures and Tables

**Table 1:** Properties of the Airy diffraction profile [Eq. (7a)]

Property	Definition	Δ*v*	*E*(Δ*v*)/*E*(0)	*f*(Δ*v*)
Sparrow limit	Δ*v* = 0.5 π	0.5 π	0.520 855	0.455 925
Area width	$\Delta v=\int_{0}^{\infty }dv{\left[\frac{2J_{1}(v)}{v}\right]}^{2}$	$\frac{16}{3\pi }=0.540\;380\pi$	0.463 082	0.506 873
Rayleigh width	Δ*v* = 0.5 Δ*v*_Airy_	0.609 835π	0.367 516	0.588 443
Airy disk, Δ*v*_Airy_	*E*(*v*) = 0	1.219 670π	0	0.837 785

**Table 2:** Properties of the Lommel Diffraction Profile [Eqs. (4a,b)] for *u* = π

Property	Definition	$\Delta v/\sqrt{u}$	*E*_rel_	$f(\Delta v/\sqrt{u})$	*E*_min_/*E*_max_
Sparrow limit	Δ*v* = 0.5π	0.886 227	0.525 145	0.380 803	0.997 362
Area width	Eq. (7)	1.023 941	0.417 900	0.458 319	0.808 131
Rayleigh width	Δ*v* = 0.5 Δ*v*_Airy_	1.498 744	0.136 878	0.647 732	0.265 165
Geometrical width	Δ*v* = π	1.772 454	0.060 510	0.698 182	0.123 190
Airy disk, Δ*v*_Airy_	$f(\Delta v/\sqrt{u})=0.837\;785$	2.997 488	0.032 497	0.837 785	0.064 741

## Footnotes

1 A paraxial approximation, cos*β* = 1, is assumed throughout this paper because this assumption is implicit in Eqs. (4a,b) and (6a). Without it, *r*_0_ and *r* would be equal to the distances *P*_0_*O* and *OP*, respectively, and inclination factors would appear in most equations.

2 Equation (6a) is approximate in that it ignores the “shrinkage” caused by the fact that the maxima of the composite pattern do not coincide exactly with the maxima of the superimposed profiles.

3 In this limit, Eq. (7a) results because one has |*α* (*u*,*v*)|^2^ → (*u*J_1_(*v*)/*v*)^2^. However, it is misleading to refer to the Fraunhofer-Airy formula as a “pinhole limit”. The aperture diameters used in pinhole photography are on the order of 0.5 mm to 1 mm and, as shown by Eqs. (2) and (5), correspond to fairly large values of *u* except for infinitely large image distances.

4 This quantity is germane to Lommel profiles and is zero in the Airy limit *u* → 0.

5 The relative central irradiance *E*_min_/*E*_max_ corresponding to this Rayleigh width is only 0.27. Alternatively the Rayleigh width of the Lommel profile can be evaluated as $\Delta v/\sqrt{u}=1.075\;287$, which is smaller than the value in Table 2 but gives the familiar relative central irradiance 0.735 932 found for Airy profiles.
